# Sustainability via Active Garden Education (SAGE): results from two feasibility pilot studies

**DOI:** 10.1186/s12889-017-4163-5

**Published:** 2017-03-10

**Authors:** Rebecca E. Lee, Nathan H. Parker, Erica G. Soltero, Tracey A. Ledoux, Scherezade K. Mama, Lorna McNeill

**Affiliations:** 1 Center for Health Promotion and Disease Prevention, College of Nursing and Health Innovation, Arizona State University, Phoenix, AZ USA; 2 Texas Obesity Research Center, Department of Health and Human Performance, University of Houston, Houston, TX USA; 3 Department of Kinesiology, College of Health and Human Development, The Pennsylvania State University, University Park, PA USA; 4 Department of Health Disparities Research, University of Texas M.D. Anderson Cancer Center, Houston, TX USA

**Keywords:** Children, Preschool, Physical activity, Fruit, Vegetables, Eating behavior, Early intervention, Curriculum, Program evaluation

## Abstract

**Background:**

Low physical activity (PA) and fruit and vegetable (F&V) consumption in early childhood are continued public health challenges. This manuscript describes outcomes from two pilot studies for Sustainability via Active Garden Education (SAGE), a program designed to increase PA and F&V consumption among 3 to 5 year old children.

**Methods:**

SAGE was developed using community-based participatory research (CBPR) and delivered to children (*N* = 89) in early care and education centers (ECEC, *N* = 6) in two US cities. Children participated in 12 one-hour sessions that included songs, games, and interactive learning activities involving garden maintenance and taste tests. We evaluated reach, efficacy, adoption, implementation, and potential for maintenance of SAGE following the RE-AIM framework. Reach was evaluated by comparing demographic characteristics among SAGE participants and residents of target geographic areas. Efficacy was evaluated with accelerometer-measured PA, F&V consumption, and eating in the absence of hunger among children, parenting practices regarding PA, and home availability of F&V. Adoption was evaluated by the number of ECEC that participated relative to the number of ECEC that were recruited. Implementation was evaluated by completion rates of planned SAGE lessons and activities, and potential for maintenance was evaluated with a parent satisfaction survey.

**Results:**

SAGE reached ECEC in neighborhoods representing a wide range of socioeconomic status, with participants’ sociodemographic characteristics representing those of the intervention areas. Children significantly increased PA during SAGE lessons compared to usual lessons, but they also consumed more calories in the absence of hunger in post- vs. pre-intervention tests (both *p* < .05). Parent reports did not suggest changes in F&V consumption, parenting PA practices, or home F&V availability, possibly due to low parent engagement. ECEC had moderate-to-high implementation of SAGE lessons and curriculum. Potential for maintenance was strong, with parents rating SAGE favorably and reporting increases in knowledge about PA and nutrition guidelines for young children.

**Conclusions:**

SAGE successfully translated national PA guidelines to practice for young children but was less successful with nutrition guidelines. High adoption and implementation and favorable parent reports suggest high potential for program sustainability. Further work to engage parents and families of young children in ECEC-based PA and nutrition programming is needed.

## Background

Low physical activity (PA) and fruit and vegetable (F&V) consumption in early childhood contribute to obesity and related health compromising conditions later in the life course [1–10]. Healthy behaviors such as physical activity (PA) [9] and dietary habits [1–4], develop in early childhood and track through youth, with weight status tracking into adulthood [1, 5–8, 10]. There are inverse associations between PA and childhood obesity across all age groups [11–14], and exposure to a healthy diet early in life can promote a lifetime of healthy dietary habits [1, 4, 8]. The 2011 US Institute of Medicine’s (IOM) Early Childhood Obesity Prevention Policies Report identified early care and education centers (ECEC) as a primary target for behavioral intervention, because most young children in the US (<5 years) are enrolled in an ECEC [15]. Among preschool aged children, the ECEC is the setting where children are the most sedentary throughout their entire day [16]. Innovative strategies are needed to increase PA and reduce sedentary time as well as to promote healthy eating habits in ECEC [17].

Garden-based education has increased in popularity with schools, families and children [18–22]. Previous work has suggested community input is important for development, implementation and sustainability of garden-based interventions for school-age children [23]. Involving stakeholders via a participatory approach can help increase the reach of intervention efforts and enhance execution of intervention protocols by promoting feasible strategies. This, in turn, can produce more robust outcomes and increase efficacy and promote long term adoption essential for broad based dissemination [22]. Similarly, results from development efforts for the intervention pilot study described herein suggest that involving ECEC staff, parents, and community members in these processes improves potential for success [24]. There has been little focus on PA in garden-based interventions among young children [22], with most existing interventions aiming to improve dietary habits with mixed success [22, 25–31]; although, one study in school aged children (grades 4 and 5, usually about age 9 and 10 years) reported that garden curricula can increase active time in school [32]. The few studies that have tested the effect of garden interventions on PA and F&V consumption neglected to evaluate factors that may impact internal and external validity (i.e., reach, efficacy, adoption, implementation, maintenance) [22]. These factors are vital to informing potential future implementation, adoption and sustainability of evidence-based interventions [33, 34].

Unlike previous interventions in ECEC [35–39], the Sustainability via Active Garden Education (SAGE) curriculum used a community-based participatory research (CBPR) approach to meet US national guidelines [15] and US ECEC accreditation standards [40]. CBPR approaches are inherently ecologic, incorporating voices from community members, practitioners, and policy makers. Thus, this study was guided by the Ecologic Model of Physical Activity (EMPA) [41, 42], which posits that micro-level environmental settings like the ECEC can create opportunities for PA and for F&V consumption that can directly determine day-to-day choices. The EMPA further suggests dynamic linkages such that health promotion efforts in ECEC may produce secondary impacts transferring to the home environment (and vice versa) via exo-environmental linkages (e.g., greater parent awareness, home availability of F&V owing to child requests based on experiences in ECEC) [43, 44]. These exo-environmental linkages eventually influence macro-level policies at the center and community level that can reduce health disparities [43, 44].

This study tested the efficacy of the SAGE intervention in two pilot projects and evaluated the internal and external validity of the pilot tests using the RE-AIM framework. SAGE was developed using a CBPR strategy over 6 years [24] and was piloted as a 12-session, garden-based, feasibility trial implemented in ECEC to increase PA and F&V consumption [45]. Following a RE-AIM strategy [33, 34], we developed and tested the reach, efficacy, adoption, implementation and potential for maintenance of SAGE in two pilot studies in two cities. Investigation of efficacy-related outcomes was hypothesis-driven, whereas investigation of reach, adoption, implementation and potential for maintenance outcomes was exploratory. Reach was defined as the representativeness of our sample of participating children. We evaluated the efficacy of SAGE on improving PA, F&V intake, and hunger and fullness cues among children, as well as PA parenting practices and the home availability of F&V. It was hypothesized that SAGE would increase PA, F&V intake, and response to hunger and fullness cues among children in ECEC and improve PA parenting practices and home availability of F&V outside of ECEC. Adoption was measured by the number of eligible sites that participated, and implementation of sessions delivered. The potential for maintenance was evaluated with parent surveys.

## Methods

### Participants and intervention design

SAGE was implemented in the US by trained research staff in four ECEC in Houston, Texas (SAGE 1) and in two ECEC in Phoenix, Arizona (SAGE 2). All centers were licensed and accredited offering full and half day care to preschool aged children. All students between the ages of 3–5 who were enrolled in the preschool class at each participating center were eligible to participate. A sub-sample of parents (*N* = 20) from SAGE 2 completed a demographic survey, parenting PA practices questionnaire, and a satisfaction survey following the intervention; specific sample sizes by instrument are presented in Table 1. Unique among previous, nutrition-based curricula, SAGE was designed to source concepts from the plant life cycle as metaphors for humans growing into healthy and strong adults. This creative approach to garden-based ECEC curricula emanated from community partners and purposed to increase interest and engagement among teachers and learners. SAGE promoted, and offered opportunities for, increasing PA, reducing sedentary time, and improving knowledge of, appreciation for, and consumption of F&V. Feedback from implementers, our CAB and ECEC directors in SAGE 1 helped to fine-tune SAGE 2; thus, results are presented separately for each of the pilot studies. Detailed description of the development of the SAGE intervention protocol has been presented previously [46].

**Table 1:** Individual measurement instruments and participants’ participation

Instrument	Participant who was measured	Pilot Study	T1	During intervention	T2
Participant demographics	Parent for child and family	2	*N =* 20	-	-
Physical activity (accelerometry)	Child	1 & 2	*N* = 70	*N* = 70	-
Child vegetable and fruit consumption (non-consecutive 3-day food records)	Parent for child	1	*N =* 23	-	*N =* 10
		2	*N =* 15		*N =* 10
Recognition of hunger and fullness cues	Child	1	*N =* 25	-	*N =* 25
		2	*N =* 29		*N =* 28
Physical activity parenting practices	Parent	1	*N =* 16	-	*N =* 11
Home food availability	Parent	1	*N =* 16	-	*N =* 10
		2	*N =* 21		*N =* 13
Parent satisfaction	Parent	2	-	-	*N =* 13

Pilot Study 1 = SAGE 1 and Pilot Study 2 = SAGE 2

### Recruitment of ECEC

SAGE Pilots 1 and 2 were conducted using identical recruitment methods. ECEC within a five-mile radius of the University campus were identified. In both cases, the neighborhoods surrounding the campuses were lower to middle SES and had higher proportions of ethnic minorities residing there. In collaboration with our Partnership and CAB, a colorful and inviting postcard was developed and sent to ECEC directors. Using a standardized script, research assistants followed up with directors within 1 week with a phone call and email to describe the project. Four out of five centers contacted in Houston SAGE Pilot 1 enrolled. The director of the fifth center decided not to participate, owing to scheduling conflicts, such as field trips and teacher in-service days. Two ECEC were invited in Phoenix and agreed to participate. After sites were identified, parents were asked to participate in a parents’ night event which was used for promotional and recruitment purposes. At parents’ night, parents of children between the ages of 3 and 5 years were provided with a consent document that explained their and their child’s participation in the study. A research assistant was present to explain the informed consent form and answer any questions. Parents who were unable to attend parent’s night were informed of the study by research assistants as they dropped off or picked up their child. Once the parents understood the informed consent and their role in the study, they had the opportunity to enroll their child in the intervention.

### Intervention overview

**Fig. 1:**
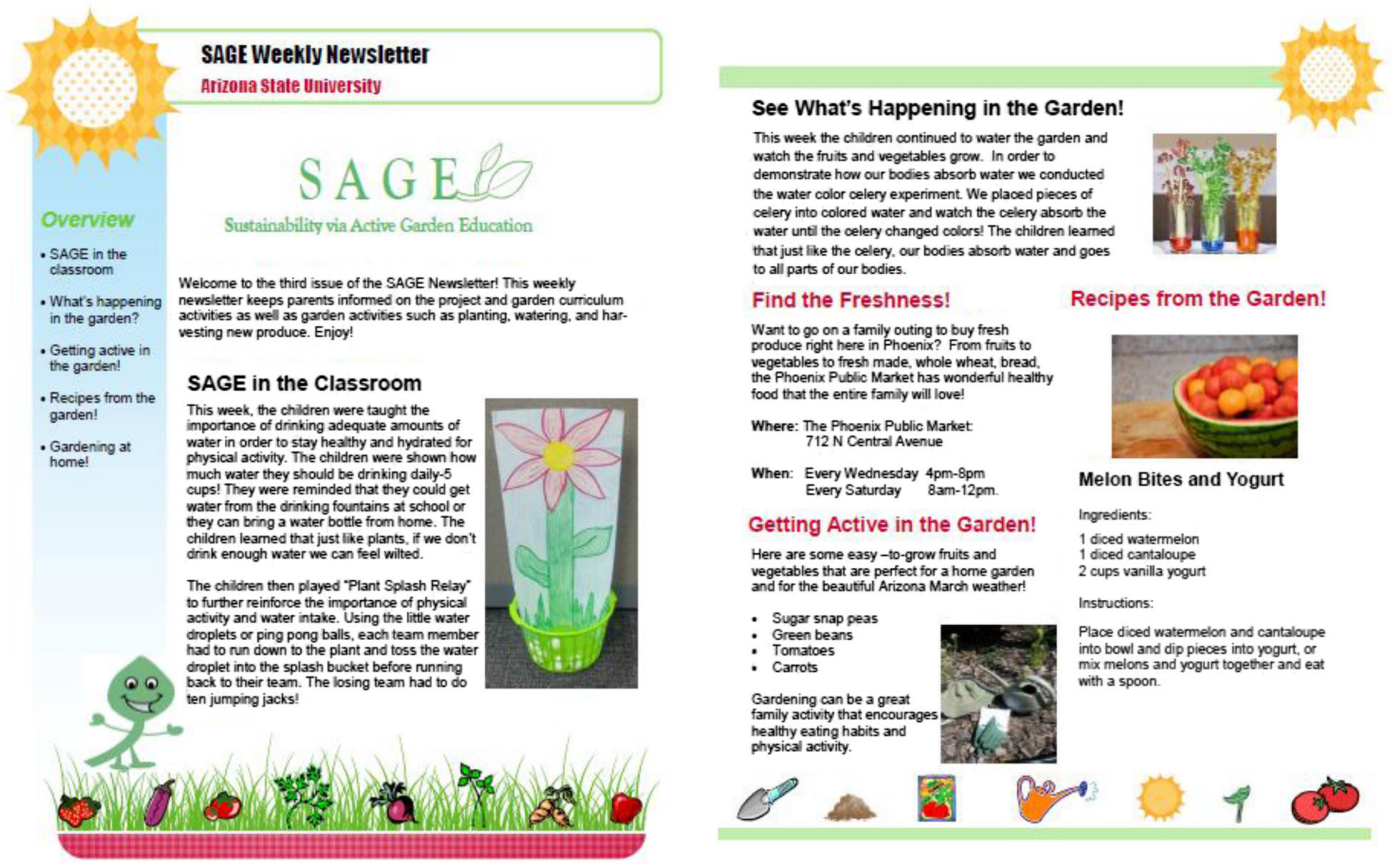
Sample SAGE Newsletter

The SAGE curriculum was implemented in participating centers twice a week in 1 hour sessions. Children participated in 12 one-hour sessions that included songs, games, and interactive learning activities involving garden maintenance and taste tests. Research assistants (i.e. undergraduate and graduate students) were paired and trained to deliver the intervention and were taught classroom management skills. All research assistants attended weekly meetings with the project director and principal investigator in order to give a report on program delivery and to discuss any challenges and potential barriers. Weekly newsletters developed in consort with our CAB were sent home with the children and used to engage parents in the program. Newsletters were available in English and Spanish and included information about what was going on in the classroom that week, to keep parents engaged and knowledgeable about SAGE. Newsletters also offered easy ideas for culturally tailored home activities and recipes, along with highlighting community resources that promoted PA and F&V consumption such as farmer’s markets or local gardening activities. Figure 1 presents a sample newsletter.

### Measures

***Reach*** of the population was measured as the sample representativeness of the population of the Census tract in which the ECEC were located. Using a written survey, parents from SAGE 2 reported on child age, gender, ethnicity and language spoken at home as well as family income (See Table 2). The decision to employ the demographic survey was made following SAGE 1; therefore, demographic data from parents whose children participated in SAGE 1 ﻿ were unavailable. From the American Community Survey, 2009–2013 5-year estimates were used to indicate neighborhood sociodemographic variables at the Census tract level for each ECEC and to determine how representative the participants whom we enrolled were of the surrounding area [47].

**Table 2:** Participant characteristics of those in SAGE Pilot 2, *N =* 20

Characteristic	*N* (%)
Ethnicity	
White	1 (5%)
African American	2 (10%)
Hispanic or Latino	13 (65%)
Asian	2 (10%)
Native American	1 (5%)
Other	1 (5%)
Country of Origin	
United States	13 (65%)
Mexico	4 (20%)
United Kingdom	2 (10%)
Language	
English	11 (65%)
Spanish	7 (35%)
More than one language	2 (10%)
Education^a^	
Less than high school	4 (20%)
High school or GED	8 (40%)
College or higher	4 (20%)
Income	
Less than $10,000	2 (10%)
$15,000–$19,000	3 (15%)
$20,000–$24,999	1 (5%)
$25,000–$29,999	2 (10%)
$30,000–$39,999	2 (10%)
$40,000–$49,999	1 (5%)
$50,000–$59,999	2 (10%)
$60,000 or more	1 (5%)
Not Reported	6 (30%)

^a^Note. Column does not total to 100% because of missing data for four participants

***Efficacy*** was measured at the child (PA, F&V, and eating in the absence of hunger), parent (parenting practices and knowledge) and home environment (F&V availability) levels.

Child *physical activity,* including light, moderate, and vigorous PA, was measured using ActiGraph GT3X accelerometers. Accelerometers were worn around the waist, centered over the right hip. During the intervention, children wore the accelerometer for 1 hour twice a week during the SAGE sessions only. SAGE staff fitted participating children with their designated devices upon arriving at ECEC for lessons, monitored device positioning throughout lessons, assisted children with realigning them as necessary, and collected devices after each lesson for data downloading and processing. Devices were initialized to collect activity at 60Hz, and light, moderate, and vigorous PA were processed in 15-second epochs according to validated cut-points for preschool aged children [48]. Specifically, light activity was defined as 800-1679 counts per minute (CPM), moderate activity as 1680-3367 CPM, and vigorous activity as at least 3368 CPM [48]. Accelerometers were initialized to start recording activity 10 min before and to continue recording activity 10 min after lessons (80 min total) to account for slight variations in lesson start and stop times. For each lesson, SAGE staff recorded the exact time at which lessons started, and, for purposes of accelerometer data processing, SAGE lessons were assumed to end 60 min after this time. Therefore, only 60 min of accelerometer-measured PA were possible for each SAGE lesson. Minutes of PA in each of the three intensities were combined, so that PA during SAGE lessons could be compared to the IOM recommendation of 15 min of total PA per hour, and sedentary (non-PA) time was calculated as the result of 60 min minus the total of light, moderate, and vigorous PA minutes. The same procedure was repeated for each of the SAGE lessons (12 in Houston, ten in Phoenix), and average minutes of activity and sedentary time per lesson attended were calculated for each child at each intervention site. Children also wore accelerometers in their ECEC for one hour the week prior to the SAGE program, during the same time at which SAGE lessons were scheduled. For example, if SAGE were due to be delivered on Tuesday at 9 am, then the pre-test would be done during the Tuesday at 9 am during the week preceding the intervention commencement. These data, processed in the same manner described above, were used to compare PA during SAGE lessons to the PA children may perform during a similar, non-SAGE hour in their ECEC.

Child *vegetable and fruit consumption* was measured with non-consecutive 3-day food records from parents, which are validated and preferred methods of dietary assessment among preschoolers [49–51]. Parents were trained to complete the food records, taught portion sizes using food models, and provided with phone support. The United States Department of Agriculture’s super tracker software was used to assess food records [52]. Research assistants inputted the data from the food records onto the software. Super tracker then generated nutrient intake reports which provided the total cups of fruits and vegetables consumed on each day that data was available. Totals were averaged across the 3 days to provide the average number of cups of fruit and vegetables consumed per day.

*Child recognition of hunger and fullness cues* was measured by adapting the laboratory Eating in the Absence of Hunger test [53] to the classroom setting to determine the degree to which children ate in the absence of physiologic hunger [2, 54]. In the adapted protocol, trained research assistants conducted the assessment following a center provided meal. Tests were done during regular SAGE sessions which varied by ECEC. At the beginning of the assessment, pretend play was used to teach concepts of hunger and fullness using three tummy dolls, one doll with a full tummy, one doll with a tummy that was just right, and one doll with an empty tummy. Children were asked to identify their level of satiety using the tummy dolls to reinforce that the full tummy doll had eaten too much, the empty tummy doll had not eaten enough, and the just right tummy doll had eaten just enough to feel energetic for play time. Children were then given two palatable, center approved snacks in pre-weighed snack bags. In SAGE 1, one snack bag contained a salty snack of pretzels (20 g, 71 kcals) and the other snack bag contained a sweet snack of unwrapped M&Ms (28 g, 136 kcals). In SAGE 2, one snack bag contained Cheezit crackers (30 g, 136.8 kcals) and the other snack bag contained animal crackers (30 g, 150 kcals).

The children were instructed to taste one piece of each snack and were asked to rate their preference for the snack using a yummy, yucky, or just okay face to ensure that each snack was considered acceptable by the children. The children were then told that they could play with the toys in the classroom or continue snacking. After 10 min, instructors collected the snack bags and brought them back to the lab for weighing.

The *Preschooler PA Parenting Practices* survey is a 17 item instrument that measures the degree to which parents encourage their child to be physically active. Responses from all items are summed with a higher score indicating parenting practices that are more supportive of physical activity. This instrument has been validated for use among parents of preschoolers, including Latino parents, and has shown moderate to excellent test-retest reliability (0.56–0.85) [55].

The *F&V Home Availability* questionnaire was used to measure F&V items in the home. This self-report questionnaire has strong internal consistency α = 0.79 among parents of preschoolers and validity with home-inventory checks with parents of 4th and 6th graders [56].

***Adoption*** was measured by calculating the percentage of ECEC that participated in SAGE from the total ECEC that were invited to participate.

***Implementation*** was defined as the number of sessions implemented and the number of activities completed per session (songs, games, hunger/fullness activity, taste testing, science experiments, and garden activity). Activities were coded as completed or not completed by research assistants delivering lessons on a formatted fidelity checklist after a sample of 63% of lessons completed in SAGE Pilot 1 and 90% of lessons completed in SAGE Pilot 2. Checklists were not completed in all sessions owing to staffing and resource limitations.

***Potential for maintenance*** was measured by a *parent satisfaction survey.* Parents completed a survey of 9 items measuring their level of satisfaction with their participation in the SAGE program and their interaction with the research team. The survey also asked parents about physical activity and nutrition knowledge gained through SAGE and the extent to which children engaged in SAGE activities in the home. Responses were recorded using a 5 point Likert scale where 1 = not satisfied and 5 = completed satisfied. See Table 3.

**Table 3:** Percent of parents that were satisfied with the SAGE program and reported an increase in health knowledge (*N* =13)

Item	n(%)
Parents satisfied with their child’s participation in the SAGE program.	12(92)
Parents satisfied with the level of communication from the project team.	8(62)
Parents satisfied with the helpfulness of the SAGE project team.	8(62)
Parents that indicated SAGE improved their child’s knowledge of nutrition.	11(85)
Parents that indicated SAGE improved their child’s knowledge of physical activity.	10(77)
Parents that indicated SAGE improved their own knowledge of nutrition.	10(77)
Parents that indicated SAGE improved their own knowledge of physical activity.	9(69)
Parents that indicated their child shared information learned in the SAGE program at home.	4(31)
Parents that indicated their child asked to do activities learned in the SAGE program, like sing songs or play games, at home.	7(54)

#### Analyses

Descriptive analyses (e.g., percentages) were used to evaluate exploratory outcomes related to reach, adoption, implementation, and potential for maintenance. Repeated measures ANOVA and ANCOVA (controlling for the ECEC each child attended as a potential covariate) were used to evaluate outcomes related to efficacy, including changes in PA from pre-intervention lessons to SAGE lessons and changes in F&V intake and eating in the absence of hunger from pre- to post-intervention. Repeated measures ANOVA and ANCOVA were also used to examine changes in home availability of fruits and vegetables and parenting physical activity practices. Differences between outcome measures were screened to insure they fit roughly normal distributions. All analyses were conducted using IBM SPSS version 22 [57], and differences were considered significant at *p* < .05.

## Results

### Reach

The ECEC in SAGE Pilot 2 were located in two separate Census tracts in Phoenix (*n* = 2) with residents from a wide range of sociodemographic backgrounds. Based on reports from parents from SAGE 2 (see Table 2; *N =* 20) children (male = 55%, female = 45%) were, on average, 3.9 (*SD =* .72) years old. The majority of parents identified as Hispanic or Latino (65%) and reported that their country of origin was the U.S. (65%). The language most predominantly spoken in the home was English (55%) with 35% reporting that Spanish was predominantly spoken in the home. Income was evenly distributed and most parents had a high school (40%) or college (20%) education. The sample favorably represented the residents of Census tracts containing ECEC, which were 52% Hispanic or Latino (*SD* = 15.5%), had 64% of adults 25 or older had completed high school or its equivalent (*SD* = 7.8%), and 44% of adults 25 or older had completed at least some college (*SD* = 9.2%). Median household income of Census tracts containing ECEC was, on average, $19,430 (*SD* = $3,665) [47]. In SAGE 1, children attended 63% of the sessions, on average (SD = 26, range = 8–100). In SAGE 2, children attended 78% of the sessions, on average (SD = 15, range = 50–100).

### Efficacy

#### Physical activity

Table 4 compares PA and sedentary time during a pre-intervention hour to PA and sedentary time during SAGE lessons for SAGE 1 and SAGE 2. Before controlling for ECEC as a potential covariate, increases in PA and decreases in sedentary time were statistically significant in both SAGE 1 and SAGE 2. In SAGE 1, mean PA increased from 8.70 min (*SD* = 5.97) in a pre-intervention hour to 14.10 min (*SD* = 4.95) during SAGE lessons [*F*(28) = 23.70, *p* < .05], and mean sedentary time decreased from 51.30 min (*SD* = 5.97) in a pre-intervention hour to 45.90 min (*SD* = 4.95) during SAGE lessons [*F*(28) = 23.70, *p* < .001]. In SAGE 2, mean PA increased from 8.62 min (*SD* = 3.99) in a pre-intervention hour to 13.31 min (*SD* = 4.35) during SAGE lessons [*F*(40) = 39.56, *p* < .001], and mean sedentary time decreased from 51.38 min (*SD* = 3.99) in a pre-intervention hour to 46.69 min (*SD* = 4.35) during SAGE lessons [*F*(40) = 39.56, *p* < .001]. Controlling for ECEC as a potential covariate, the change in PA observed in SAGE 1 was no longer statistically significant [*F*(27) = 1.65, *p* = .21], and there was no significant interaction between change in PA and ECEC [*F*(27) = .13, *p* = .72]. The change in sedentary time observed in SAGE 1 was also no longer statistically significant after controlling for ECEC as a potential covariate [*F*(27)=,1.65 *p* = .21], and there was no significant interaction between change in sedentary time and ECEC [*F*(27) = .13, *p* = .72]. In SAGE 2, the change in PA from the pre-intervention hour to during lessons remained significant after controlling for ECEC as a potential covariate [*F*(39) = 4.11, *p* = .05], but there was no significant interaction between change in PA and ECEC [*F*(39) = .08, *p* = .78]. The change in sedentary time observed in SAGE 2 also remained statistically significant after controlling for ECEC [*F*(39) = 4.11, *p* = .05], but there was no significant interaction between change in sedentary time and ECEC [*F*(39) = .08, *p* = .78].

**Table 4:** Comparison of accelerometer-measured physical activity between a pre-intervention hour and SAGE lessons

	SAGE 1 (*N* = 29)					SAGE 2 (*N* = 41)				
	Pre-intervention [mean(SD)]	SAGE lessons (n = 12) [mean(SD)]	*df*	*F*	*p*	Pre-intervention [mean(SD)]	SAGE lessons (n = 10) [mean(SD)]	*df*	*F*	*p*
Physical activity (min)	8.70 (5.97)	14.10 (4.95)	28	23.70	<.05	8.62 (3.99)	13.31 (4.35)	40	39.56	<.001
	*Controlling for ECEC*		*27*	1.65	.21	*Controlling for ECEC*		39	4.11	.05
Sedentary time (min)	51.30 (5.97)	45.90 (4.95)	28	23.70	<.05	51.38 (3.99)	46.69 (4.35)	40	39.56	<.001
	*Controling for ECEC*		27	1.65	.21	*Controlling for ECEC*		39	.08	.78

#### Dietary habits

Food records showed that children ate an average of .72 (*SD* = .58) cups of vegetables per day at baseline and an average of .77 (*SD* = .19) cups of vegetables per day after participating in SAGE; however, this increase was not significant before (*F*(1, 10) = .072, *p* > .05) or after controlling for center (*F* (1,0) = .065, *p* > .05). Food records also showed that children ate an average of .99 (*SD* = .87) cups of fruit per day at baseline and an average of 1.15 (*SD* = .76) cups of fruit per day after participating in SAGE. Similarly, the increase in fruit consumption was not significant before (*F*(1,10) = .343, *p* > .05) or after controlling for center (*F* (1,9) = .002, *p >* .05). Table 5 presents information on child dietary habits, parents and home environment.

**Table 5:** Child eating habits and parent and home outcomes pre- and post-intervention in SAGE pilot studies

	SAGE Pilot 1 (*N* = 29)		SAGE Pilot 2 (*N* = 41)	
	Pre-Intervention M (SD)	Post-Intervention M (SD)	Pre-Intervention M (SD)	Post-Intervention M (SD)
Fruit and vegetable intake				
Vegetable Intake (cups)	.73 (.60)	.75 (.20)	.70 (.63)	.78 (.21)
Fruit Intake (cups)	.99 (.36)	1.33 (.81)	.98 (1.31)	.93 (.71)
Eating in the Absence of Hunger (kCals)	80.63 (60.54)	102.03 (63.32)	55.33 (70.57)	86.39 (56.29)
Parenting Physical Activity Practices	-	-	59.0 (7.02)	63.0 (5.32)
Home availability of F&V				
Vegetables (servings)	11.83 (2.48)	12.83 (2.79)	6.27 (4.56)	6.36 (4.15)
Fruits (servings)	13.17 (4.92)	13.5 (5.72)	12.36 (8.79)	12.73(8.21)

#### Eating in the absence of hunger

Kilocalories consumed in the absence of hunger significantly increased from pre to post-intervention. The average number of kilocalories consumed in the absence of hunger at baseline was 68.51 (*SD =* 66.07) and the average number of kilocalories consumed in the absence of hunger post-intervention was 94.54 *(SD =* 59.94). This increase was statistically significant before (*F*(1,47) = -7.162, *p* = .01); however, the increase in kilocalories consumed was not significant after controlling for center (*F*(1,46) = .002, *p* > .05).

#### Parent physical activity practices

The Preschooler Physical Activity Parenting Practices (PPAPP) survey was used to measure parenting practices that encouraged PA in SAGE 2 parents. The average PPAPP score at baseline was *M* = 57.3 (*SD* =8.73) and *M* = 62.64 (*SD* =4.32) at post-intervention. The increase in parenting practices that encouraged PA was not significant before (*F*(1,6) = 4.603, *p* = .076) or after controlling for center (*F*(1,5) =4.051, *p* > .05).

#### Home food environment

Parents reported an average of 8.24 (*SD* =4.74) servings of vegetables available in the home at baseline and an average of 8.65 (*SD* =4.83) servings of vegetables available in the home after participating SAGE; this increase was not significant before (*F*(1,16) = .536, *p* > .05) or after controlling for center (*F*(1,15) = .437, *p* > .05). Parents reported an average of 12.65 (*SD* =7.48) servings of fruit available in the home at baseline and an average of 13.0 (*SD* =7.25) servings of fruit available in the home after participating in SAGE; this increase was also not significant before (*F*(1,16) = .202, *p* > .05) or after controlling for center (*F*(1,15) = .464, *p* > .05).

### Adoption

Four of five invited ECEC participated in Houston SAGE Pilot 1, and both of the invited ECEC participated in Phoenix SAGE Pilot 2 suggesting high rates of adoption (86%).

### Implementation

Implementation was moderate to high. Each center delivered at least ten of the 12 SAGE lessons, suggesting good implementation (83%). Class size varied greatly as some centers were larger than others. Smaller centers averaged 3–7 children per session and larger centers averaged 10–19 children per session.

In Houston, SAGE Pilot 1, across all ECEC lessons, seven out of ten songs (70.0%), 40 out of 49 possible games (81.6%), 20 out of 30 hunger and fullness activities (86.7%), 29 out of 30 taste tests (96.7%), seven out of nine science experiments (77.8%) and 20 out of 21 garden activities (95.2%) were completed. In Phoenix in SAGE Pilot 2 across all ECEC lessons, ten out of 11 songs (90.9%), 46 out of 49 possible games (93.9%), 17 out of 18 hunger and fullness activities (94.4%), 18 out of 18 taste tests (100.0%), three out of four science experiments (75.0%) and 13 out of 13 garden activities (100.0%) were completed.

### Potential for maintenance

#### Parent perceptions

Reports from parent satisfaction surveys indicated that almost all (92%) parents were satisfied with their child’s participation in SAGE. The majority of parents were satisfied with the level of communication (62%) and helpfulness (62%) from the SAGE team. Most parents believed that participating in SAGE improved their child’s knowledge of PA (77%) and nutrition (85%). The majority of parents also believed that participating in SAGE improved their own knowledge of PA (69%) and nutrition (77%). Parents reported that 31% of children shared information learned from SAGE in the home and a little over half of the children requested to do activities learned in SAGE (54%), like sing songs or play games, at home. Parent survey responses are presented in Table 3.

## Discussion

This study presents the findings from the SAGE Pilot tests and demonstrates that a PA and F&V garden-based education program for ECEC can achieve good reach, implementation, and efficacy in increasing PA as well as demonstrating high potential for maintenance. Study recruitment was well received with high levels of participation and good representation of underserved communities, and strong implementation. PA almost doubled during SAGE lesson time compared to regular lesson time, approaching IOM recommended amounts suggesting high efficacy. In contrast, the SAGE Pilot studies were less successful at improving dietary habits. As well, although popular with parents, parenting practices and home environments did not show significant change as a result of child participation in SAGE.

SAGE showed good efficacy for increasing PA by demonstrating that garden-based education can significantly increase PA in the ECEC, the place where young children are typically at their least active throughout their entire waking day [16]. This compares favorably with one study done in schools which showed increases in PA in older children participating in a school garden program [32]. On average, SAGE’s one hour lessons, offered about twice a week, approached the IOM recommendation of at least 15 min of activity during waking hours [15]. In SAGE 1, the increase in PA from pre-intervention lessons to lessons during the intervention was no longer significant after controlling for ECEC. This may reflect differences in the regularly-scheduled activities taking place in centers during pre-intervention lesson PA measurement, or differences in ECEC indoor or outdoor spaces that influenced PA during intervention activities. In contrast, SAGE demonstrated poor efficacy in enhancing eating behaviors in children, consistent with one other garden-based study in ECEC [58]. Although there was a small increase, it was not what had been anticipated from several previous school-based studies [27–30]. It may be that the intervention did not provide sufficient dose or duration to change eating behaviors, although the SAGE intervention was more intensive than a previous study [58]. Possibly parents may have perceived that the additional emphasis on fruit and vegetables during ECE time could compensate for emphasis in improving dietary quality at home. The current results may also result from relatively low compliance with dietary assessment records from parents that resulted in a small sample size. This was designed as a small pilot study, and future work is needed to determine how to increase compliance with assessment methods, or alternative assessment strategies that reduce participant burden should be used.

Children’s eating in the absence of hunger actually increased post intervention which was the opposite of what we expected. As has been previously reported, we used an adapted protocol suitable for a community setting [54]. Although this pioneering work has helped to lead the science in the assessment of eating in the absence of hunger in young children, the adaptation met with barriers. The team was unable to ensure that children had eaten the meal, struggled with finding appropriate snacks (complying with school rules and accommodating food allergies) and faced teaching complex concepts of hunger and fullness, which may have impacted results. We received anecdotal feedback from our initial pilot test (SAGE Pilot 1) from our CAB, ECEC directors and parents to change the snack stimulus. Future work is needed to overcome these barriers in community settings [54].

The SAGE Pilot tests were unable to detect whether parenting practices focused on PA promotion had increased, although the direction of trend suggested that it might be happening. This interpretation is consistent with the parent survey questions that suggested that SAGE was well liked, and parents believed that children’s knowledge had increased. At the same time, no changes in home availability of F&V were detected. Both of these measures suffered from low response rates from parents. As noted above, resources for better engagement and assessment strategies are needed. Methodological limitations in SAGE Pilots 1 and 2 include the potential for recall and favorability bias from parents on surveys that were used to evaluate efficacy and potential for maintenance outcomes. Further, it will be important to collect parents’ demographic data from all participating ECEC and to collect accelerometer data from children during larger samples of pre- and post-SAGE lessons.

Despite limitations in the ability to detect efficacy, there was very high adoption of the SAGE Pilots, because nearly all of the ECEC that were invited participated. High rates of implementation showed good fidelity to the curriculum. Differences in implementation variation between the two studies were due to a myriad of factors which included class size variability, time constraints, variation between the two pilot studies as well as the natural unpredictability of young children necessitating a flexible approach to curriculum delivery. Additional reports previously published suggested that SAGE was well received by ECEC staff who believed it easy and fun to implement (Soltero EG, Parker NH, Mama SK, Ledoux TA, Lee RE: Implementation and adoption of Sustainability via Active Garden Education (SAGE): Lessons learned from early care and education centers, forthcoming). ECEC staff also reported that SAGE complemented their existing curricula and filled a need for stronger health education. SAGE reached a representative population of the local neighborhoods. Those parents who did respond to parent surveys were very pleased with their child’s participation in SAGE. Taken together the strong reach, adoption, and implementation suggest the potential for maintenance of SAGE. Future investigations should examine longer term efficacy and adoption of the SAGE program, including training teachers and staff to implement lessons.

The SAGE Pilot tests demonstrate that it is possible to increase PA among young children in the ECEC with active programming, and ECEC staff and parents are interested and pleased with this type of programming. Additional strengths include a rigorous development protocol, a larger sample size than previously published studies of ECEC (*N* = 6) in two US cities, a carefully crafted and theoretically grounded intervention strategy relying on a participatory approach that involved the people whom the intervention served. SAGE relied on a thorough assessment and valid and reliable measures, although it suffered from low sample size on some measures. SAGE was also low cost and innovative. Limitations included an inability to detect efficacy on nutrition and parenting related outcomes, probably a result of low sample size. Important measures necessary to establish reach were not collected or available, and better planning is needed in future studies to document reach appropriately. Although all children who were present participated in the curriculum, we were only able to document those that had parent consent. In addition to children without informed consent, it is also likely that siblings and other family members may have benefited from the SAGE curriculum. These exo-level ecological linkages are fodder for future investigations. Few parents returned survey measures that had been sent home with children. Qualitative interviews suggested much higher parent interest, in part a result of efforts made to foster parent engagement (e.g., newsletters, announcements at parent night), suggesting more work should be done to engage parents and reduce the burden of completing questionnaires [46].

## Conclusions

Results suggest that SAGE is an important first step in meeting PA recommendations and improving health education in ECEC in the US. SAGE demonstrated good reach, efficacy, adoption, implementation and high potential to be sustained in the ECEC setting, and reflects careful development involving stakeholders. Future research should test the sustainability of SAGE by continuing to work with ECEC staff, providing resources for ongoing technical support and continued efforts at engaging parents. SAGE was designed as a transcultural intervention, useful for improving early child health across a variety of cultures and communities via a long term participatory process. As a result of this highly engaged process, SAGE provides innovative and fun, garden-based programming that translates policy guidelines into practice to achieve increases in PA in preschool aged children with high internal and external validity suggesting high translatability to other cultural contexts.

## Abbreviations

ECEC: Early care and education center
F&V: Fruits and vegetables
PA: Physical activity
SAGE: Sustainability via Active Garden Education
